# Consideration of Clozapine in Patients With Treatment Resistant Schizophrenia

**DOI:** 10.1192/bjo.2024.603

**Published:** 2024-08-01

**Authors:** Niema Moazzami, Aimee Devereux

**Affiliations:** Bradford District Care Trust, Bradford, United Kingdom

## Abstract

**Aims:**

The aim of the audit is to measure the performance of Bradford District Care Trust (BDCT) against the NICE guideline's quality standard: ‘Adults with schizophrenia that has not responded adequately to treatment with at least 2 antipsychotic drugs are offered clozapine.’

**Methods:**

We identified the 273 patients prescribed clozapine in BDCT as of January 2023. 202 had been prescribed clozapine for more than 5 years and these were excluded. Of the remaining 71, 34 were excluded for reasons such as their diagnosis i.e. Parkinson's or personality disorder or inadequate information within the clinical record. The final sample consisted of 37 patients prescribed clozapine within the last 5 years with a diagnosis of treatment resistant schizophrenia (TRS).

To define an ‘adequate trial’ of an antipsychotic, an adequate duration and adequate dose was determined. It is widely recommended in literature that an adequate trial of antipsychotic should last at least 6 weeks and this time constraint was utilised for the audit. The Maudsley Guidelines minimum effective dose table was utilised for establishing adequate doses. For antipsychotics not included in this list the British National Formulary (BNF) maintenance doses were used. A data collection tool was then developed that allowed for retrospective collection of key information relating to the objectives outlined above.

**Results:**

Clozapine was offered at the appropriate time [this includes where clozapine was considered but was not felt to be suitable/was contraindicated/declined by the patient] in 13 cases (35%). In 24 cases (65%) clozapine was not offered at the appropriate time. For 21 patients there was a delay in offering clozapine after 2 adequate trials of antipsychotic medication had been given. Delays ranged between 9 days and 15 years, with the average [mean] delay being 3.7 years. There were 3 patients who were initiated on clozapine without the completion of 2 adequate trials of other antipsychotic medication.

**Conclusion:**

In summary, this audit measured BDCT's adherence to the NICE guideline on clozapine for TRS. Of the 37 patients in the final sample, 35% received clozapine at the appropriate time, while 57% experienced delays with an average delay of 3.7 years. Notably, 8% commenced clozapine without completing the recommended antipsychotic trials. These results emphasize the importance of improving adherence to the guideline, as delays in offering clozapine pose potential risks for patients with TRS. Ensuring timely access to this treatment is crucial, as it has the potential to significantly enhance patient outcomes.

